# AI-based spectroscopic monitoring of real-time interactions between SARS-CoV-2 and human ACE2

**DOI:** 10.1073/pnas.2025879118

**Published:** 2021-06-14

**Authors:** Sheng Ye, Guozhen Zhang, Jun Jiang

**Affiliations:** ^a^School of Artificial Intelligence, Anhui University, Hefei, Anhui 230601, People’s Republic of China;; ^b^Gusu Laboratory of Materials, Suzhou, Jiangsu 215123, People’s Republic of China;; ^c^Hefei National Laboratory for Physical Sciences at the Microscale, Chinese Academy of Sciences Center for Excellence in Nanoscience, School of Chemistry and Materials Science, University of Science and Technology of China, Hefei, Anhui 230026, People’s Republic of China

**Keywords:** SARS-CoV-2, IR spectroscopy, neural networks, protein dynamics

## Abstract

The novel coronavirus, severe acute respiratory syndrome coronavirus 2 (SARS-CoV-2), invades a human cell via human angiotensin-converting enzyme 2 (hACE2) as the entry, causing the severe coronavirus disease (COVID-19). The interactions between hACE2 and the spike glycoprotein (S protein) of SARS-CoV-2 hold the key to understanding the molecular mechanism to develop treatment and vaccines, yet the dynamic nature of these interactions in fluctuating surroundings is very challenging to probe by those structure determination techniques requiring the structures of samples to be fixed. Here we demonstrate, by a proof-of-concept simulation of infrared (IR) spectra of S protein and hACE2, that time-resolved spectroscopy may monitor the real-time structural information of the protein−protein complexes of interest, with the help of machine learning. Our machine learning protocol is able to identify fine changes in IR spectra associated with variation of the secondary structures of S protein of the coronavirus. Further, it is three to four orders of magnitude faster than conventional quantum chemistry calculations. We expect our machine learning protocol would accelerate the development of real-time spectroscopy study of protein dynamics.

## Significance

The COVID-19 caused by SARS-CoV-2 virus has posed a tremendous threat to human health. The interactions between human angiotensin-converting enzyme 2 and the spike glycoprotein of SARS-CoV-2 hold the key to understanding the molecular mechanism to develop treatment and vaccines. However, the simulation of these interactions in fluctuating surroundings is challenging because it requires many electronic structure calculations at the quantum mechanics level for a large number of representative configurations. We report a machine learning protocol that can efficiently predict the IR spectra of SARS-CoV-2 with high efficiency and characterize fine changes in IR spectra associated with variations of protein secondary structures. Machine learning provides a cost-effective tool for monitoring of real-time interactions between the SARS-CoV-2 and human ACE2.

The ongoing pandemic of COVID-19, a highly infectious disease caused by severe acute respiratory syndrome coronavirus 2 (SARS-CoV-2), has posed tremendous threat to human health and well-being by having affected tens of millions of people and killed more than 1 million affected since December 2019 (1). It has spurred enormous efforts in biological and biomedical research to search for a solution to this fatal disease, which rapidly advance our knowledge about it, including the identity of the pathogen (i.e., SARS-CoV-2), the genome sequence of the virus, and the structural basis for coronavirus recognition and infection (2–5). SARS-CoV-2 recognizes human angiotensin-converting enzyme 2 (hACE2) as the entry receptor to host cells using its surface spike glycoprotein (S protein) (1). The interactions of S protein with hACE2 have been subjected to intensive investigations by several groups (6–10), which laid the foundation for comprehensive understanding of the invasion of SARS-CoV-2 into the human body at the atomic scale (11), helps the search for intermediate hosts of the coronavirus (12), and will guide the design of therapeutics and vaccines (11, 13). Since the physiological environment in which S protein and hACE2 interact is always fluctuated due to the dynamic nature of water, a dynamic picture of the interactions between them is needed for precise mechanistic understanding that will inspire modulation and application (14). Unfortunately, such information relies on real-time tracking of protein conformations, which cannot be achieved by powerful structure characterization techniques with atomic precision like X-ray diffraction and cryoelectron microscopy, because they require fixed structures in samples. It motivates us to develop alternative approaches to resolve the issue.

Recently, time-resolved infrared (IR) spectroscopy techniques have realized successful monitoring of changes of secondary structure with time (15), signaling the feasibility of real-time observation of protein dynamics in ambient conditions using spectroscopy. However, to facilitate the monitoring of specific peptide fragments in a secondary structure typically requires isotope labeling (e.g., C=O in the amide of protein backbone is replaced with ^13^C=O or C=^18^O) in the preparation of samples, which is, unfortunately, tedious and expensive for systematic investigation on conformation changes in protein dynamics. Therefore, it is desirable to develop isotope labeling-free spectroscopy to accelerate structure study of proteins for biological and biomedical sciences. To achieve this goal, one needs to employ quantum chemistry calculations to complete spectra signal assignment and structure determination. In fact, it relies on computer simulations of various possible conformers to nail the job, which is, unfortunately, very expensive for macromolecules like proteins. One of the biggest bottleneck problems in spectroscopic measurement of proteins is lack of rapid theoretical interpretation that can timely translate spectra signals into structural information. As a result, it is nearly impossible for an experimental spectroscopic study to monitor continuous structural changes associated with protein functions. Developing a cost-effective spectra simulation protocol is a pressing task to advance the real-time spectroscopy study of protein structures.

Machine learning (ML), a collection of statistics-based methods which gain prediction power from the learning of big data, has emerged as a powerful toolkit to reduce the barrier to revealing the structure−property relationship (16). It has been increasingly popular in the study of molecules and materials, such as predicting chemical reaction routes (17) and accelerating discovery of materials (18). Especially, neural networks (NN), a subclass of ML algorithms, are well recognized for handling complex nonlinear problems. NN established a predictive model for desired properties by iterative optimization of a complex high-dimensional function in a virtually infinite space of parameters. This feature makes it a transferrable tool for predicting protein spectra (19).

In this article, we developed and applied a cost-effective ML protocol, to predict the IR spectra along with the kinetic process of a COVID-2019 virus (SARS-CoV-2) protein binding to hACE2. The efficient simulation of IR signals of different states of the coronavirus associated with the changes in its secondary structure is very encouraging for studying dynamic interactions between S protein of SARS-CoV-2 and human ACE2 with the help of ML techniques. This will enable a real-time spectroscopic monitoring of protein structure evolution for this deadly virus, providing valuable information for understanding its molecular mechanism, as well as developing cures and vaccines. ML should provide a cost-effective tool for simulating optical properties of SARS-CoV-2.

## Results and Discussion

**Fig. 1.**
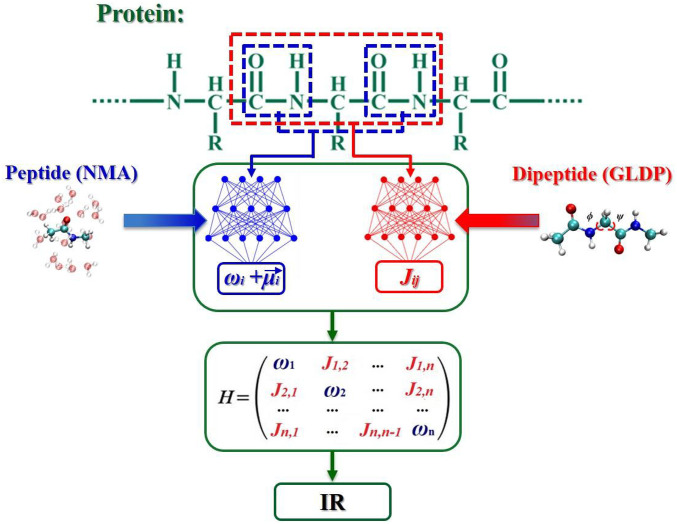
ML protocol for the IR spectra of proteins.

The technique details of this ML protocol have been elaborated elsewhere (20). Here we just sketch the basic idea of the framework (Fig. 1). We adopt a divide-and-conquer strategy to treat the amide I vibrations of the whole protein. The vibration of a protein is represented as a set of *n* oscillators associated with each peptide bond in its backbone. The Frenkel exciton model is employed to construct a vibrational model Hamiltonian (21), in which the diagonal elements are the frequency (*ω*_*i*_) of the *i*th amide I oscillator, and the off-diagonal elements include the coupling coefficient (*J*_*ij*_) between two oscillators *i* and *j* (Fig. 1). To obtain these matrix elements, a protein is split into individual peptide bonds and dipeptides. The values of *ω*_*i*_ and $\vec{\mu_{i}}$ are predicted from an NN model of peptide, that is, *N*-methylacetamide (22, 23). For off-diagonal elements, there are two scenarios: Those coupling coefficients between two neighboring oscillators are computed using an NN model of dipeptide, that is, *N*-acetyl-glycine-*N*′-methylamide (GLDP) (24, 25); those between a pair of nonneighboring oscillators are calculated with the dipole approximation (26) assuming that, given the distances between oscillators are greater than the length of the peptide bond,$J_{ij}=\left(1/4\pi \varepsilon_{o}\right)\left[\left(\vec{\mu_{i}}\cdot \vec{\mu_{j}}/r_{ij}^{3}\right)-3\left(\left(\vec{\mu_{i}}\cdot \vec{r_{ij}}\right)\left(\vec{\mu_{j}}\cdot \vec{r_{ij}}\right)/r_{ij}^{5}\right)\right]$, where *ε*_0_ is the dielectric constant, $\vec{\mu_{i}}$ ($\vec{\mu_{j}}$) is the transition dipole of peptide bond *i* (*j*), and *r*_*ij*_ is the vector connecting dipoles *i* and *j*. After all matrix elements of the model Hamiltonian are obtained, IR spectra are simulated using the SPECTRON program developed by Mukamel and coworkers (27). We also make this ML protocol available online to facilitate the development of experimental spectroscopy of rapid protein IR spectroscopy prediction (28).

**Fig. 2.**
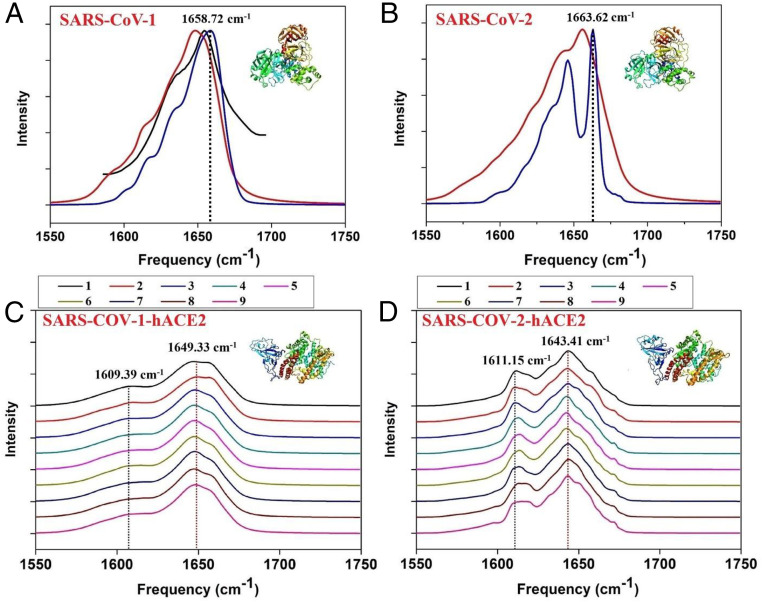
ML-predicted IR spectra of SARS-CoV-1, SARS-CoV-2, SARS-CoV-1-hACE2, and SARS-CoV-2-hACE2. (*A*) Comparison of experimental (30) (black line) and ML-predicted (red line: single crystal structure [PDB ID code 2AMQ]; blue line: average of 1,000 configurations) spectra of SARS-CoV-1. (*B*) ML-predicted IR spectra of SARS-CoV-2 based on a single crystal structure (red lines, PDB ID code 6LU7) and 2,000 MD configurations (blue lines). (*C*) ML-predicted IR spectra of SARS-CoV-1-hACE2 (PDB ID code 2AJF) during 10us MD simulation (contains nine trajectories; 1,000 snapshots for nos.1 to 8 trajectories, 334 snapshots for no. 9 trajectory). (*D*) Same as *C* but for SARS-CoV-2-hACE2 (PDB ID code 6M17). Intensity is scaled to have the same maximum intensity for each panel.

We first simulated the amide I IR spectra of SARS-CoV-1 and SARS-CoV-2 using the ML protocol described in Fig. 1 by the averages from 1,000 and from 2,000 snapshots, respectively (which would be prohibitively expensive via direct quantum mechanics computations). The simulation environment was water, which serves as the solvent of protein solution. The real protein solution contains more than protein and water molecules, but the specific aim in this work is to investigate how our ML protocol accelerates the simulation of protein IR spectra to facilitate the atomic-scale understanding of structure changes associated with S protein of SARS-CoV-2 binding to hACE2, not to understand impacts of specific enviromental factors in solution on protein−protein complex. Therefore, we made a necessary simplification of the protein solution model and considered water as the only component other than protein of interest in our model. For this reason, we used molecular dynamics (MD) simulation trajectories of protein solutions which only involve water as the environment. The structures and trajectories of SARS-CoV-1 and SARS-CoV-2 are obtained from MD simulations by ourselves and Komatsu et al. (29), respectively. The good agreement of SARS-CoV-1 between our ML predictions (average 1,000 snapshots) and experimental spectra (30) is evident from the high Spearman rank correlation coefficients (*ρ* = 0.93) (31) (Fig. 2), which was widely used to measure the agreement between the predicted and experimental spectra. From the 10 microseconds (μs) MD simulation trajectories (contain 10 trajectories; 1,000 snapshots for nos. 1 through 10 trajectories) obtained from Komatsu et al., we have chosen the amide I IR spectra of the SARS-CoV-2 with this ML protocol by average 2,000 snapshots in the first 2us for comparison, since the results have converged on the considered number of snapshots (*SI Appendix*, Figs. S1 and S2) (for the results of the remaining 8,000 snapshots, please see *SI Appendix*, Fig. S1). Then we predicted the amide I IR spectra of the SARS-CoV-2 with this ML protocol (average 2,000 snapshots). As shown in Fig. 2, the dominant peak of SARS-COV-2 has a 5 cm^−1^ blue shift compared with SARS-COV-1 (SARS-COV-1: 1,658.72 cm^−1^, SARS-COV-2: 1,663.62 cm^−1^). This may be accounted for by SARS-COV-2 having a larger portion of the β-turns content than SARS-COV-1 (Table 1), and β-turns possessing an amide IR signal of higher frequency (32–34). Importantly, our ML protocol identified the fine difference in amide I IR spectra associated with the difference between their secondary structures, and it is four orders of magnitude faster than conventional quantum chemistry calculations (Table 1).

**Table 1.** Average secondary structure content (computed by Stride program) of various coronaviruses and comparison of the time required for computing IR spectra of a single structure by Density Functional Theory (DFT) and our ML model in the framework of vibrational exciton model

	β-Strands (%)	β-Turns (%)	α-Helix (%)	310-Helices (%)	Coil (%)	Bridge (%)	DFT (s)	ML (s)
SARS-COV-1	30.1	19.9	23.9	2.5	21.0	2.5	1,165,320	70.69
SARS-COV-2	28.3	25.5	20.3	2.6	20.4	2.9	1,173,000	72.68
SARS-CoV-1-hACE2	7.6	23.2	45.2	3.9	18.0	2.2	1,482,120	100.80
SARS-CoV-2-hACE2	7.0	21.2	45.6	3.2	21.8	1.2	1,474,440	98.68
Trimeric SARS-CoV-2 S protein (closed state)	30.7	25.6	18.0	1.9	21.9	1.7	6,068,100	5,295.60
Trimeric SARS-CoV-2 S protein (open state)	30.6	25.1	18.7	1.9	22.1	1.6	6,068,100	4,613.40
RBD/hACE2 binding (S1 state)	32.3	22.1	9.4	7.8	27.7	0.8	370,440	20.64
RBD/hACE2 binding (S2 state)	31.8	21.5	12.1	6.2	27.3	1.2	370,440	20.64
RBD/hACE2 binding (S3 state)	33.5	25.5	12.1	6.2	21.5	1.2	370,440	20.64
RBD/hACE2 binding (S4 state)	33.0	21.4	9.4	7.8	27.3	1.2	370,440	20.64
RBD/hACE2 binding (S5 state)	33.0	21.9	11.6	4.7	27.6	1.2	370,440	20.64

All reported times refer to calculations on an eight-core Intel(R) Xeon(R) CPU (E5-2683v4 at 2.1 GHz). DFT, Density Functional Theory.

Then we simulated the amide I IR spectra of SARS-CoV-1-hACE2 (hACE2 in complex with the receptor binding domain of spike protein from SARS-CoV-1) and SARS-CoV-2-hACE2 (hACE2 in complex with the receptor binding domain of spike protein from SARS-CoV-2) by average 8,334 snapshots with our ML protocol (Fig. 2). These MD simulation data were retrieved from the website of D. E. Shaw Research (35). Each MD simulation is 10 μs and contains nine trajectories (1,000 snapshots for nos. 1 to 8 trajectories, 334 snapshots for no. 9 trajectory). We also chose the averaged IR spectra of the first trajectory (1st: 1,200 ns which contains 1,000 snapshots) for comparison. From the average secondary structure content analysis (by average 1,000 snapshots from no. 1 trajectory) by the Stride program (36), the random coil content of RBD2-hACE2 was higher than that of RBD1-hACE2, and the β-turn content was lower than that of RBD1-hACE2, which led to a 6 cm^−1^ red shift of the dominant peak (32–34, 37) (RBD1-hACE2: 1,649.33 cm^−1^; RBD2-hACE2: 1,643.41 cm^−1^) (Table 1). Again, the difference in secondary structures between RBD1-hACE2 and RBD2-hACE2 is clearly characterized by our ML-based IR spectra simulation.

**Fig. 3.**
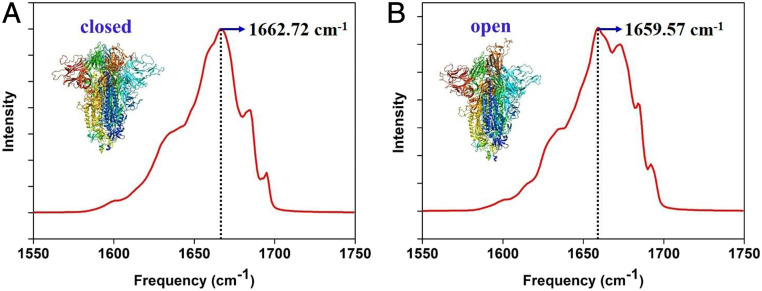
ML-predicted IR spectra of Trimeric SARS-CoV-2 S protein. (*A*) Closed state (PDB ID code 6VXX). (*B*) Open state (PDB ID code 6VYB).

The trimeric SARS-CoV-2 S protein has two distinctive states: closed state and open state (6). Intriguingly, they have substantially different secondary structures. From the 10 μs MD simulation trajectories (contain nine trajectories; 1,000 snapshots for nos. 1 to 8 trajectories, 334 snapshots for no. 9 trajectory) obtained from the website of D. E. Shaw Research, we have simulated the amide I IR spectra of the trimeric SARS-CoV-2 S protein with closed and open states by using 800 snapshots in the first trajectory for comparison. (For the results of remain trajectories, please see *SI Appendix*, Fig. S3.) It is noticed that the dominant peak of the trimeric SARS-CoV-2 S protein in the open state has a 3 cm^−1^ red shift compared with closed state, which coincides with the secondary structure content difference (the β-turn of the open state is lower but the coil content is higher than closed state (33, 37, 38) (Fig. 3 and Table 1).

**Fig. 4.**
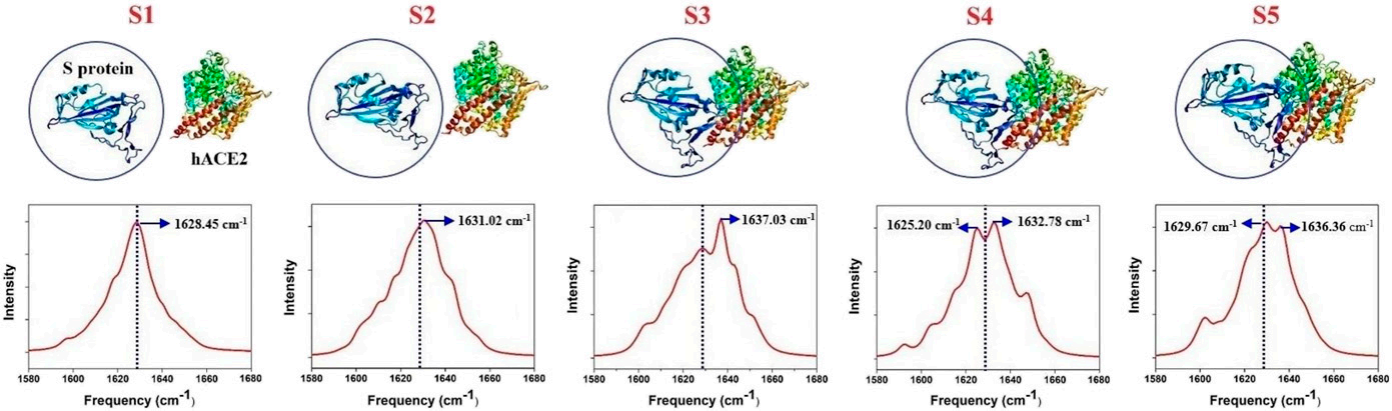
Five representative states of the receptor-binding domain of the SARS-CoV-2 spike (S protein) and the human ACE2 (hACE2) receptor were selected from the combination trajectory.

Finally, we investigated the dynamics of S protein of SARS-CoV-2 interacting with hACE2 interaction, using our ML protocol. Five representative structures were selected from D. E. Shaw Research (35). We predicted the IR spectra of S protein in different states during the combination process by ML and calculated the average secondary structure components in each state (Fig. 4 and Table 1). The identified five states are of chemical interest for understanding the process of dynamic interaction between the S protein of SARS-CoV-2 and the hACE2. They are five successive states used for describing such a process. Specifically, we have identified S1 to S5 states based on the trajectory of accelerated weighted ensemble MD simulations (source: D. E. Shaw Research) of 9,072 ps duration. Specifically, S1 denotes *t* = 0 ps in the MD simulation; S2: *t* = 1,008 ps; S3: *t* = 3,931.2 ps; S4: *t* = 4,838.4 ps; and S5: *t* = 7,056 ps. From the S1 to S2 state, the IR spectra has a 2.57 cm^−1^ blue shift. The analysis of the average secondary structure content showed that the main change from S1 to S2 was the increased content of α-helix which led to a blue shift (33, 37, 38). From S2 to S3, the IR spectra also has a 6 cm^−1^ blue shift corresponding to the averaged secondary structure content change (33, 37, 38) (S2 to S3: β-turns increased while coil decreased). From S3 to S4, the IR spectra has a 5 cm^−1^ red shift which is caused by the β-turns and α-helix decreasing while coil content increased (32, 34, 37, 38). From S4 to S5, the IR spectra has a 4 cm^−1^ blue shift which is caused by β-turns and α-helix increasing (33, 34). The changes in the IR spectra of the S protein under different states associated with the changes in the secondary structure are correctly captured by our ML protocol. We have further investigated the amide I signals of different SARS-CoV-2 spikes (S proteins), as shown in *SI Appendix*, Fig. S4; from Sa to Sb, the dominant peak of spectra has a blue shift, which corresponds to the increase of β-turns and α-helix and the simultaneous decrease of coil (*SI Appendix*, Table S1). From Sb to Sc, the dominant peak of spectra has a red shift, which corresponds to the decrease of β-turns and α-helix and the simultaneous increase of coil (*SI Appendix*, Table S1). The structural change is clearly captured by the change of spectra (*SI Appendix*, Fig. S4). This supplementary result suggests that our ML protocol can help spectroscopy experiments track structural changes of proteins; we think our method provides a promising route for studying real-time dynamics regarding to the interactions of SARS-CoV-2 and human ACE2.

## Conclusions

In conclusion, we have proposed a cost-effective ML protocol for predicting amide I IR spectra of SARS-COV-2 spike protein. The change in secondary structure of coronavirus can be clearly captured by our ML protocol, indicating its potential for monitoring of real-time interactions between SARS-CoV-2 and human ACE2. ML technique significantly accelerates the simulation of IR spectra of protein complexes, crucial for developing time-resolved IR spectroscopy techniques for studying dynamic protein−protein interactions.

## Methods

MD simulations for SARS-CoV-1 (PDB ID code 2AMQ) were performed with the GROMACS package (39) and the OPLS-AA force fields (40). Electrostatic interactions were treated by the Particle mesh Ewald method, and Coulomb interactions were truncated at 12.0 Å. Energy minimization was performed for 50,000 cycles for each protein. Thereafter, an equilibration process in isothermal-isobaric (NPT) ensemble with an integration time step of 2 fs ran for 0.5 ns (40). Production dynamics were performed for a period of 2 ns in the NPT ensemble at 300 K while maintaining pressure at 1 atm. One thousand configurations were extracted with a 2-ps interval for calculating the IR spectra.

## Supplementary Material

Supplementary File

## Data Availability

All study data are included in the article and *SI Appendix*. All Protein Data Bank (PDB) ID code information is mentioned in the article (2AMQ, 6LU7, 2AJF, 6M17, 6VXX, 6VYB).

## References

[1] P. Zhou ., A pneumonia outbreak associated with a new coronavirus of probable bat origin. Nature 579, 270–273 (2020). 32015507 10.1038/s41586-020-2012-7 PMC7095418

[2] N. Zhu ., A novel coronavirus from patients with pneumonia in China, 2019. N. Engl. J. Med. 382, 727–733 (2020). 31978945 10.1056/NEJMoa2001017 PMC7092803

[3] R. Lu ., Genomic characterisation and epidemiology of 2019 novel coronavirus: Implications for virus origins and receptor binding. Lancet 395, 565–574 (2020). 32007145 10.1016/S0140-6736(20)30251-8 PMC7159086

[4] F. Wu ., A new coronavirus associated with human respiratory disease in China. Nature 579, 265–269 (2020). 32015508 10.1038/s41586-020-2008-3 PMC7094943

[5] D. Wrapp ., Cryo-EM structure of the 2019-nCoV spike in the prefusion conformation. Science 367, 1260–1263 (2020). 32075877 10.1126/science.abb2507 PMC7164637

[6] R. Yan ., Structural basis for the recognition of SARS-CoV-2 by full-length human ACE2. Science 367, 1444–1448 (2020). 32132184 10.1126/science.abb2762 PMC7164635

[7] J. Lan ., Structure of the SARS-CoV-2 spike receptor-binding domain bound to the ACE2 receptor. Nature 581, 215–220 (2020). 32225176 10.1038/s41586-020-2180-5

[8] J. Shang ., Structural basis of receptor recognition by SARS-CoV-2. Nature 581, 221–224 (2020). 32225175 10.1038/s41586-020-2179-y PMC7328981

[9] A. C. Walls ., Structure, function, and antigenicity of the SARS-CoV-2 spike glycoprotein. Cell 181, 281–292.e6 (2020). 32155444 10.1016/j.cell.2020.02.058 PMC7102599

[10] Q. Wang ., Structural and functional basis of SARS-CoV-2 entry by using human ACE2. Cell 181, 894–904.e9 (2020). 32275855 10.1016/j.cell.2020.03.045 PMC7144619

[11] H. Zhang, J. M. Penninger, Y. Li, N. Zhong, A. S. Slutsky, Angiotensin-converting enzyme 2 (ACE2) as a SARS-CoV-2 receptor: Molecular mechanisms and potential therapeutic target. Intensive Care Med. 46, 586–590 (2020). 32125455 10.1007/s00134-020-05985-9 PMC7079879

[12] Z. Liu ., Composition and divergence of coronavirus spike proteins and host ACE2 receptors predict potential intermediate hosts of SARS-CoV-2. J. Med. Virol. 92, 595–601 (2020). 32100877 10.1002/jmv.25726 PMC7228221

[13] M. Zheng, L. Song, Novel antibody epitopes dominate the antigenicity of spike glycoprotein in SARS-CoV-2 compared to SARS-CoV. Cell. Mol. Immunol. 17, 536–538 (2020). 32132669 10.1038/s41423-020-0385-z PMC7091851

[14] D. E. Gordon .; QCRG Structural Biology Consortium; Zoonomia Consortium, Comparative host-coronavirus protein interaction networks reveal pan-viral disease mechanisms. Science 370, eabe9403 (2020). 33060197 10.1126/science.abe9403 PMC7808408

[15] J. Seo ., An infrared spectroscopy approach to follow β-sheet formation in peptide amyloid assemblies. Nat. Chem. 9, 39–44 (2017). 27995915 10.1038/nchem.2615

[16] K. T. Butler, D. W. Davies, H. Cartwright, O. Isayev, A. Walsh, Machine learning for molecular and materials science. Nature 559, 547–555 (2018). 30046072 10.1038/s41586-018-0337-2

[17] M. H. S. Segler, M. Preuss, M. P. Waller, Planning chemical syntheses with deep neural networks and symbolic AI. Nature 555, 604–610 (2018). 29595767 10.1038/nature25978

[18] S. Ma, S.-D. Huang, Z.-P. Liu, Dynamic coordination of cations and catalytic selectivity on zinc–chromium oxide alloys during syngas conversion. Nat. Catal. 2, 671–677 (2019).

[19] S. Ye ., A neural network protocol for electronic excitations of *N*-methylacetamide. Proc. Natl. Acad. Sci. U.S.A. 116, 11612–11617 (2019). 31147467 10.1073/pnas.1821044116 PMC6575560

[20] S. Ye ., A machine learning protocol for predicting protein infrared spectra. J. Am. Chem. Soc. 142, 19071–19077 (2020). 33126795 10.1021/jacs.0c06530

[21] P. Hamm, M. Zanni, Concepts and Methods of 2D Infrared Spectroscopy (Cambridge University Press, 2011).

[22] L. Wang, C. T. Middleton, M. T. Zanni, J. L. Skinner, Development and validation of transferable amide I vibrational frequency maps for peptides. J. Phys. Chem. B 115, 3713–3724 (2011). 21405034 10.1021/jp200745r PMC3274961

[23] T. Hayashi, W. Zhuang, S. Mukamel, Electrostatic DFT map for the complete vibrational amide band of NMA. J. Phys. Chem. A 109, 9747–9759 (2005). 16833288 10.1021/jp052324l

[24] T. la Cour Jansen, A. G. Dijkstra, T. M. Watson, J. D. Hirst, J. Knoester, Modeling the amide I bands of small peptides. J. Chem. Phys. 125, 44312 (2006). 16942147 10.1063/1.2218516

[25] T. Hayashi, S. Mukamel, Vibrational-exciton couplings for the amide I, II, III, and A modes of peptides. J. Phys. Chem. B 111, 11032–11046 (2007). 17725341 10.1021/jp070369b

[26] S. Krimm, Y. Abe, Intermolecular interaction effects in the amide I vibrations of polypeptides. Proc. Natl. Acad. Sci. U.S.A. 69, 2788–2792 (1972). 4507602 10.1073/pnas.69.10.2788 PMC389645

[27] W. Zhuang, D. Abramavicius, T. Hayashi, S. Mukamel, Simulation protocols for coherent femtosecond vibrational spectra of peptides. J. Phys. Chem. B 110, 3362–3374 (2006). 16494351 10.1021/jp055813u PMC2775088

[28] Machine Learning for Protein Spectroscopy. http://dcaiku.com:12880/platform/first. Accessed 15 April 2020.

[29] T. S. Komatsu ., COVID-19 related trajectory data of 10 microseconds all atom molecular dynamics simulation of SARS-CoV-2 dimeric main protease. Mendeley Data. https://data.mendeley.com/datasets/vpps4vhryg/2. Accessed 21 April 2020.

[30] W. Surya, M. Samsó, J. Torres, “Structural and functional aspects of viroporins in human respiratory viruses: Respiratory syncytial virus and coronaviruses” in Respiratory Disease and Infection-A New Insight, M. Vats, Ed. (InTechOpen, 2013), pp. 47–76.

[31] N. A. Besley, J. D. Hirst, Theoretical studies toward quantitative protein circular dichroism calculations. J. Am. Chem. Soc. 121, 9636–9644 (1999).

[32] R. I. Litvinov, D. A. Faizullin, Y. F. Zuev, J. W. Weisel, The α-helix to β-sheet transition in stretched and compressed hydrated fibrin clots. Biophys. J. 103, 1020–1027 (2012). 23009851 10.1016/j.bpj.2012.07.046 PMC3433599

[33] A. Barth, Infrared spectroscopy of proteins. Biochim. Biophys. Acta (BBA) Bioenerg. 1767, 1073–1101 (2007). 10.1016/j.bbabio.2007.06.004 17692815

[34] C. R. Baiz, A. Tokmakoff, Structural disorder of folded proteins: Isotope-edited 2D IR spectroscopy and Markov state modeling. Biophys. J. 108, 1747–1757 (2015). 25863066 10.1016/j.bpj.2014.12.061 PMC4390782

[35] D. E. Shaw Research, Molecular dynamics simulations related to SARS-CoV-2. D. E. Shaw Research Technical Data. https://www.deshawresearch.com/resources_sarscov2.html. Accessed 29 April 2020.

[36] D. Frishman, P. Argos, Knowledge-based protein secondary structure assignment. Proteins 23, 566–579 (1995). 8749853 10.1002/prot.340230412

[37] D. Usoltsev, V. Sitnikova, A. Kajava, M. Uspenskaya, Systematic FTIR spectroscopy study of the secondary structure changes in human serum albumin under various denaturation conditions. Biomolecules 9, 359 (2019). 10.3390/biom9080359 PMC6723850 31409012

[38] M. Kyriakidou ., An FT-IR spectral analysis of the effects of γ-radiation on normal and cancerous cartilage. In Vivo 30, 599–604 (2016). 27566078

[39] D. Van Der Spoel ., GROMACS: Fast, flexible, and free. J. Comput. Chem. 26, 1701–1718 (2005). 16211538 10.1002/jcc.20291

[40] F. S. Husseini, D. Robinson, N. T. Hunt, A. W. Parker, J. D. Hirst, Computing infrared spectra of proteins using the exciton model. J. Comput. Chem. 38, 1362–1375 (2017). 27868210 10.1002/jcc.24674 PMC5434914

