# Supplementary material for: AI-based spectroscopic monitoring of real-time interactions between SARS-CoV-2 and human ACE2
Source: Proc Natl Acad Sci U S A. 2021 Jun 14;118(26):e2025879118. doi: 10.1073/pnas.2025879118 (PMC8256048; doi:10.1073/pnas.2025879118)
Supplement: Supplementary File [file pnas.2025879118.sapp.pdf]

# Supporting Information

## AI-based Spectroscopic Monitoring of Real-time Interactions between SARS-CoV-2 and Human ACE2

Sheng Ye[a,b,c,1], Guozhen Zhang[c,1], Jun Jiang[c,2]

[a]School of Artificial Intelligence, Anhui University, Hefei, Anhui 230601, P. R. China

[b]Gusu Laboratory of Materials, Suzhou, Jiangsu 215123, P. R. China

[c]China Hefei National Laboratory for Physical Sciences at the Microscale, Chinese Academy of Sciences Center for Excellence in Nanoscience, School of Chemistry and Materials Science, University of Science and Technology of China, Hefei, Anhui 230026, People's Republic of China

*Corresponding author. E-mail: jiangj1@ustc.edu.cn

# Table of Contents

Computational Details....................2
Molecular Dynamics Simulations....................2
Criteria of the snapshots chosen and the time steps of the obtained snapshots....................3
ML-predicted IR spectra of SARS-CoV-2 during 10us MD simulation....................4
ML-predicted IR spectra of Trimeric SARS-CoV-2 spike glycoprotein in closed state and open state. 5
ML-predicted IR spectra of different S proteins....................6
References....................6

# Computational Details

# Machine learning protocol

**Neural networks architecture.** The neural networks architecture was implemented in TensorFlow (1) to predict the properties ($\omega_i$, $\vec{\mu_i}$, $J_{ij}$) f from the geometric Coulomb Matrix (CM) descriptors of the NMA and part of GLDP molecules. The NN consists of one input layer, three hidden layers and one output layer, and the number of hidden layer neurons are 32, 64 and 128, respectively. The Rectified Linear Unit activation function (2) and Adam optimizer (3) were used for NN training. The L2 regularization (4) technology was used to prevent overfitting problems in NN training. We optimized the Hyperparameters by using random search algorithms (5) in TensorFlow to create a reasonable ML protocol.

## Molecular Dynamics Simulations

**SARS:** Molecular dynamics simulations for SARS-CoV-1 (PDB Code:2amq) were performed with the GROMACS package (6) and the OPLS-AA force fields (7). Periodic boundary conditions were imposed on a central cell containing one SARS-CoV-1 and 35056 TIP3P water molecules. Electrostatic interactions were treated by the Particle mesh Ewald method and Coulomb interactions were truncated at 12.0 Å. Energy minimization was performed for 50,000 cycles for each protein. Thereafter an equilibration process in NPT ensemble with an integration timestep of 2 fs ran for 0.5 ns (7). Production dynamics were performed for a period of 2 ns in the NPT ensemble at 300K while maintaining pressure at 1 atm. 1000 configurations were extracted with a 2 ps interval for calculating the IR spectra.

The Stride program carries out the secondary structure content analysis according to the following algorithm (8):
"the weighted contributions of both the secondary structure forming hydrogen bonds (characterized by hydrogen bond energies) and the backbone torsion angles (characterized by $\phi$, $\psi$ values) are adopted to calculate percentages of $\alpha$-helices and $\beta$-strands in a protein. A unified threshold for these quantities for all types of hydrogen bonded patterns is used for precise tuning of recognition parameters. Crystallographers' assignments as provided in hundreds of available coordinate sets are used systematically for tuning the thresholds in the recognition procedure."

## Criteria of the snapshots chosen and the time steps of the obtained snapshots

The criteria of the snapshots chosen and the time steps of the obtained snapshots were stated as below:

(1) For the snapshots of SARS-CoV-1 were chosen from 2 ns MD simulation, in total 1000 configurations were extracted with a 2 ps time step interval for calculating the IR spectra.

(2) For the snapshots of SARS-CoV-2 were chosen from 10 us MD simulation, in total 10000 configurations were extracted with a 1 ns time step interval for calculating the IR spectra.

(3) For the snapshots of SARS-CoV-1-ACE2 were chosen from 10 us MD simulation, in total 8334 configurations were extracted with a 1.2 ns time step interval from start time 1200ps – 10us for calculating the IR spectra.

(4) For the snapshots of SARS-CoV-2-ACE2 were chosen from 10 us MD simulation, in total 8334 configurations were extracted with a 1.2 ns time step interval from start time 1200ps – 10us for calculating the IR spectra.

(5) For the snapshots of Trimeric SARS-CoV-2 spike glycoprotein (closed state) were chosen from 10 us MD simulation, a total of 8334 configurations were extracted with an 1.2 ns interval from start time 1200ps – 10us (contains 9 trajectories, 1000 snapshots for No.1-8 trajectories, 334 snapshots for No.9 trajectory). We chose the first 800 snapshots in each of No.1-8 trajectories (6400 in total) to calculate the IR spectra.

(6) For the snapshots of Trimeric SARS-CoV-2 spike glycoprotein (opened state) were chosen from 10 us MD simulation, a total of 8334 configurations were extracted with a 1.2 ns interval from start time 1200ps – 10us(contains 9 trajectories,1000 snapshots for No.1-8 trajectories, 334 snapshots for No.9 trajectory). We chose the first 800 snapshots in each of No.1-8 trajectories (6400 in total) to calculate the IR spectra.

## ML-predicted IR spectra of SARS-CoV-2 during 10us MD simulation

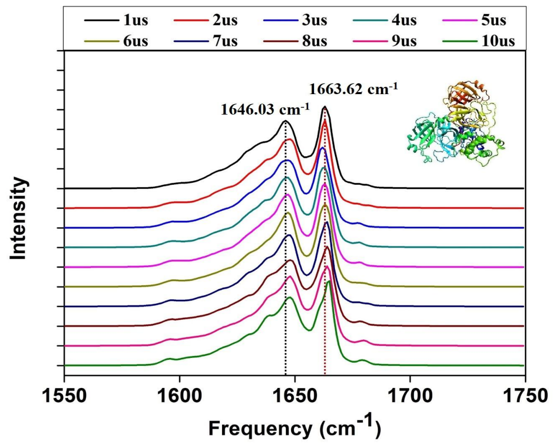


**Fig. S1**. ML-predicted IR spectra of SARS-CoV-2 (PDB: 6LU7) during 10us MD simulation (Each trajectories contains 1000 snapshots).

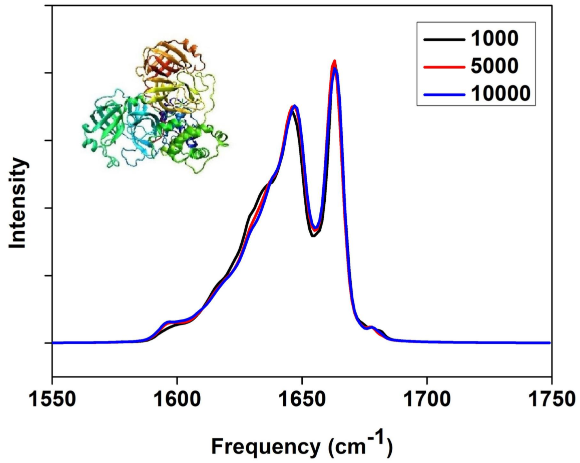


**Fig. S2**. Amide I IR spectra of the SARS-CoV-2 by computed average with 1000 (black line), 5000 (red line) and 10000 (blue line) snapshots.

## ML-predicted IR spectra of Trimeric SARS-CoV-2 spike glycoprotein in closed state and open state

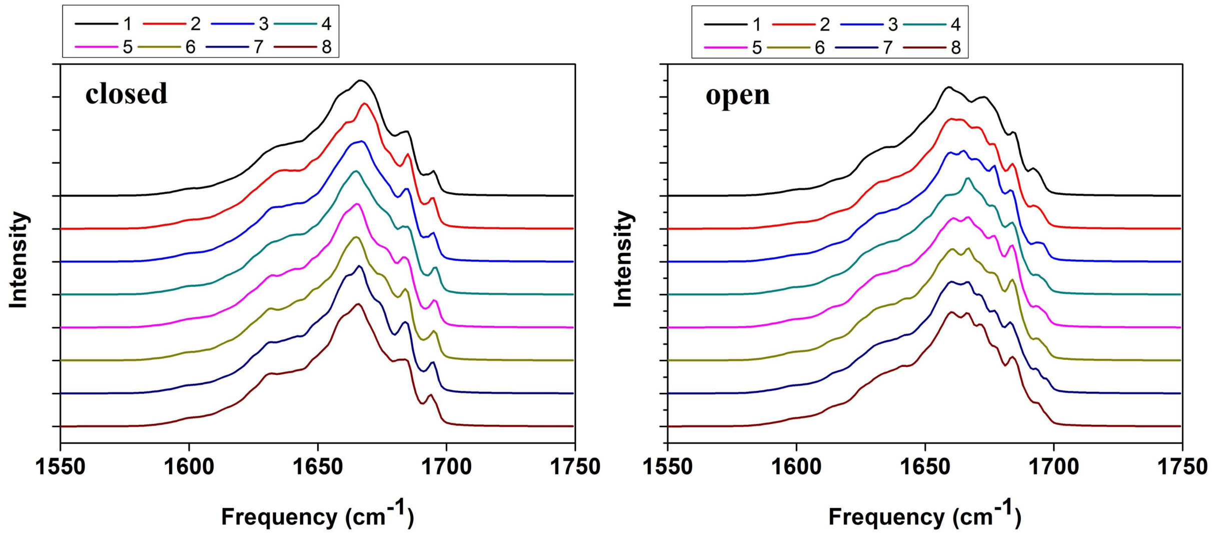


**Fig. S3**. ML-predicted IR spectra of Trimeric SARS-CoV-2 spike glycoprotein in closed state and open state (Each trajectories contains 800 snapshots).

## ML-predicted IR spectra of different S proteins

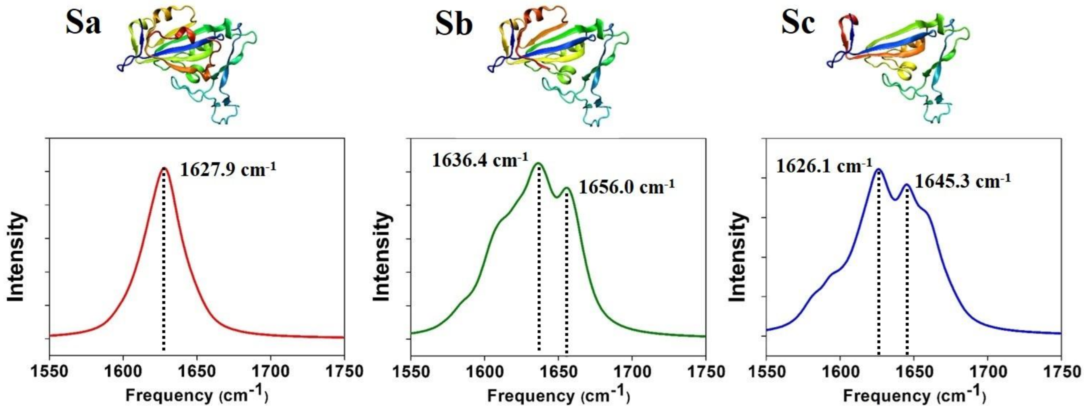


**Fig. S4**. Amide I IR spectra of S protein (Sa), and S protein missing part of the skeleton (Sb and Sc). All the Lorentzian bandwidths were 8 $cm^{-1}$.

**Table S1.** The secondary structure content (computed using Stride) of different S protein.

| Content / State | $\beta$-strands | $\beta$-turns | $\alpha$-helix | $3_{10}$-helices | Coil |
|---|---|---|---|---|---|
| a | 32.3% | 22.1% | 9.4% | 7.8% | 27.7% |
| b | 34.3% | 23.3% | 10.6% | 5.2% | 26.4% |
| c | 34.7% | 21.1% | 8.0% | 6.3% | 29.5% |

## References


1. Abadi M, *et al.* (2016) Tensorflow: A system for large-scale machine learning. *12th {USENIX} Symposium on Operating Systems Design and Implementation ({OSDI} 16)*, pp 265-283.
2. Maas AL, Hannun AY, & Ng AY (2013) Rectifier nonlinearities improve neural network acoustic models. *Proc. icml*, p 3.
3. Kingma DP & Ba J (2014) Adam: A method for stochastic optimization. *arXiv preprint arXiv:1412.6980*.
4. Ng AY (2004) Feature selection, L 1 vs. L 2 regularization, and rotational invariance. *Proceedings of the twenty-first international conference on Machine learning*, p 78.
5. Bergstra J & Bengio Y (2012) Random search for hyper-parameter optimization. *J Mach Learn Res* 13(1):281-305.
6. Van Der Spoel D*, et al.* (2005) GROMACS: fast, flexible, and free. *J. Comput. Chem.* 26(16):1701-1718.

7. Husseini FS, Robinson D, Hunt NT, Parker AW, & Hirst JD (2017) Computing infrared spectra of proteins using the exciton model. *J. Comput. Chem.* 38(16):1362-1375.
8. Frishman D & Argos P (1995) Knowledge - based protein secondary structure assignment. *Proteins: Structure, Function, and Bioinformatics* 23(4):566-579.
